# Review of a new bone tumor therapy strategy based on bifunctional biomaterials

**DOI:** 10.1038/s41413-021-00139-z

**Published:** 2021-03-16

**Authors:** Jinfeng Liao, Ruxia Han, Yongzhi Wu, Zhiyong Qian

**Affiliations:** State Key Laboratory of Oral Diseases, National Clinical Research Centre for Oral Diseases, State Key Laboratory of Biotherapy and Cancer Center, West China Hospital, West China Medical School, Sichuan University, Chengdu, Sichuan P.R. China

**Keywords:** Bone cancer, Bone cancer

## Abstract

Bone tumors, especially those in osteosarcoma, usually occur in adolescents. The standard clinical treatment includes chemotherapy, surgical therapy, and radiation therapy. Unfortunately, surgical resection often fails to completely remove the tumor, which is the main cause of postoperative recurrence and metastasis, resulting in a high mortality rate. Moreover, bone tumors often invade large areas of bone, which cannot repair itself, and causes a serious effect on the quality of life of patients. Thus, bone tumor therapy and bone regeneration are challenging in the clinic. Herein, this review presents the recent developments in bifunctional biomaterials to achieve a new strategy for bone tumor therapy. The selected bifunctional materials include 3D-printed scaffolds, nano/microparticle-containing scaffolds, hydrogels, and bone-targeting nanomaterials. Numerous related studies on bifunctional biomaterials combining tumor photothermal therapy with enhanced bone regeneration were reviewed. Finally, a perspective on the future development of biomaterials for tumor therapy and bone tissue engineering is discussed. This review will provide a useful reference for bone tumor-related disease and the field of complex diseases to combine tumor therapy and tissue engineering.

## Introduction

Bone tumors involve the invasion of tumors into bone tissue and are classified as either primary tumors or metastatic tumors. Osteosarcoma is a well-known primary malignant bone tumor that often occurs in children and adolescents. It has been reported that this disease has become the second leading cause of tumor-related death in young teenagers.^1^ The majority of patients die from lung metastases. Its annual incidence worldwide is ~1–3 cases per million.^2^ The clinical signs of osteosarcoma are not obvious without spontaneous fracture or severe pain early on. Therefore, this disease is not easily diagnosed, but the tumors grow quickly. As a result, osteosarcoma causes a large bone defect and limitations in motion and can metastasize to the lungs.^3^ The etiology of osteosarcoma is still not clear.^4^ To date, the most common clinical treatment methods for bone tumors include chemotherapy, wide surgical resection, and radiotherapy.^5^ However, osteosarcoma is not sensitive to radiotherapy and is prone to chemotherapy resistance. Surgical resection often fails to completely remove the tumor, which is the main cause of postoperative recurrence and metastasis. Moreover, osteosarcoma invades large areas of bone, which cannot repair itself, and has serious effects on the quality of life of patients.^6^ The 5-year survival rate of patients with osteosarcoma is ~60%.^7^ Unfortunately, advances in osteosarcoma treatment have reached a plateau over the past 40 years.^8^

Metastatic bone tumors start somewhere else in the body and then spread to bone tissue at a later stage. Bone tissue is one of the most common metastatic sites, and certain cancers, such as breast, prostate, colon, and lung cancer, are closely related to bone metastasis.^9–13^ Bone metastasis results from tumor cells migrating and adhering to the bone, thus interfering with the balance of bone formation and bone resorption. Osteosarcoma and bone metastasis share some similarities,^14^ but metastatic bone tumors exist in the later stage of the tumor. The primary tumor is usually diagnosed before it metastasizes to the bone after treatment. In tumor-induced bone defects, metastatic bone tumors and osteosarcoma share similar tumor niches and microenvironments. Innovative and efficient therapeutic strategies are urgently needed to solve the problems in the treatment of bone tumors.^15^

Along with the development of bionanotechnology, new innovative treatment options have been designed for bone tumor therapy. Bone tumor therapy combines the complex issues of tumor therapy and bone regeneration, which demand functional biomaterials for treatment. It is challenging to design novel strategies with the dual capabilities of both preventing tumor recurrence and supporting bone formation, demanding an interdisciplinary research background.^16,17^ Many researchers worldwide have focused their efforts on solving these bone tumor treatment problems. Although they are only in the early stages of development, new treatment methods have brought great hope to finding a cure for bone tumors.

Traditional postoperative bone tumor treatment is chemotherapy. However, these chemical drugs can lead to systemic side effects such as liver dysfunction, heart toxicity, and bone marrow suppression. The development of new supplementary or alternative tumor treatment methods based on biomaterials can avoid these side effects by selective delivery.^18–26^ Specifically, photothermal therapy is an emerging treatment method that converts near-infrared (NIR) light into localized thermal energy to destroy tumor tissue.^27–35^ Photothermal therapy is based on nanomaterials with strong NIR absorption, such as gold nanoparticles,^36–41^ carbon nanomaterials,^42,43^ magnetic nanoparticles,^44–48^ and copper nanomaterials.^49,50^ Photothermal therapy is suitable for localized tumor therapy due to the concentrated irradiation region of the laser and its ability to limit the deep penetration of heat without damaging other organs or tissues.^51–55^ With its rapid development, photothermal therapy is a potential supplement to preclinical and clinical tumor therapy. For example, photothermal therapy based on gold nanoshells has shown a great therapeutic effect in clinical trials for prostate tumor therapy.^56,57^ Photothermal therapy is a suitable candidate method for bone tumor treatment, and related studies have focused on it.

**Fig. 1:**
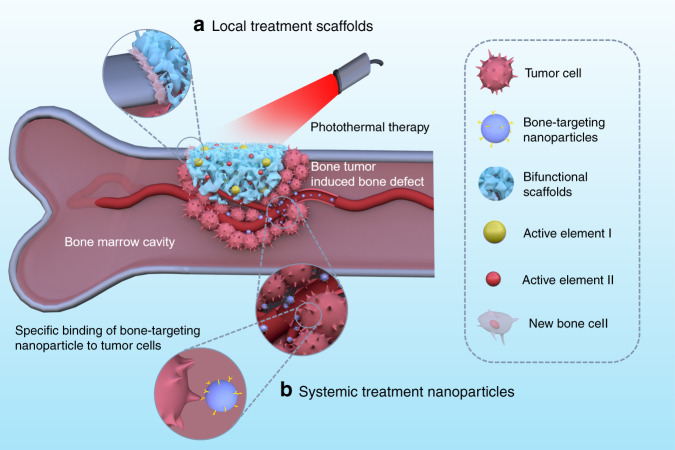
Bifunctional biomaterials include (**a**) local treatment scaffolds (such as 3D-printed scaffolds, nano/microparticle-containing scaffolds, and hydrogels) and (**b**) systemic treatment nanoparticles (such as bone-targeting nanoparticles) for tumor photothermal therapy and bone regeneration

Due to the offensive spreading of tumors into bone, bone metabolism becomes unbalanced. Healthy bone tissue is resorbed and invaded by the tumor, leading to bone defects. After tumor therapy, these bone defects become the next issue of concern. Bone tissue engineering is a fascinating field that gives hope to bone regeneration. The biomaterial scaffolds developed for bone tissue regeneration include nanofibers, 3D-printed scaffolds, hydrogels, microspheres, and nanoparticles.^58–64^ Bioactivity, biocompatibility, and biodegradability are critical concerns in scaffold design, playing an important role in bone regeneration.^65–68^ In particular, the key parameters of porosity, stiffness, and viscoelasticity can regulate cell adhesion, cell proliferation, and osteogenesis differentiation.^69–76^ Scaffolds provide cells with sustainable regenerative factors, provide physical and biological support, and mobilize stem cells to regenerate the defect cavity.^77–82^ Bifunctional scaffolds have been designed in recent years, thanks to the tireless work of researchers, for tumor photothermal therapy and bone repair. These scaffolds are capable of simultaneously providing tumor therapy and enhanced bone regeneration, a useful “two birds, one stone” strategy. Figure 1 shows a bone tumor that was killed by bifunctional biomaterials through either local or systemic administration. The locally administered bifunctional scaffolds (such as 3D-printed scaffolds, nano/microparticle-containing scaffolds, and hydrogels) were inserted into the bone defect area for tumor photothermal therapy and, subsequently, improved bone repair. The systemically administered nanoparticles penetrated blood vessels to target the bone tissues for tumor treatment and to inhibit bone reabsorption. Some representative examples of bifunctional biomaterials were summerized and listed in Table 1.

**Table 1:** Examples of bifunctional biomaterials mainly include 3D-printed scaffolds, nano/microparticle-containing scaffolds, hydrogels, and bone-targeting nanomaterials in tumor therapy and bone regeneration

Platform	Biomaterial	Tumor therapy	Bone regeneration	Ref.
3D-printed scaffolds	3D-printed Fe-CaSiO_3_ scaffold	Synergistic photothermal and ROS therapy enhanced Saos-2 bone tumor therapy in the backs of nude mice	The active elements Fe, Ca, Si in the scaffold enhanced large bone defect repair in rabbits	^99^
	Silicone resin-derived larnite/C 3D-printed scaffold	Photothermal effect inhibited MNNG/HOS human osteosarcoma tumor growth in nude mice	Promotion of bone formation in critical-sized rat calvarial defect by stimulating an osteogenesis-related gene	^101^
	Coloaded CaO_2_ and Fe_3_O_4_ nanoparticle 3D-printed biomaterial	Reactive oxygen species therapy and magnetic hyperthermia produced a synergistic effect in MNNG/HOS osteosarcoma tumor-bearing BALB/c nude mice	Ca^2+^ ions released from CaO_2_ nanoparticles improved the bone regeneration in SD rats cervical defects	^102^
Nano/microparticle-containing scaffolds	SrFe_12_O_19_ nanoparticle modified-mesoporous bioglass/chitosan porous scaffold	Photothermal therapy in MG-63 bone tumors	Bone regeneration in a critical calvarial defect model	^110^
	Chitosan matrix incorporated Fe_3_O_4_ nanoparticles and GdPO_4_ nanorods	Photothermal effects to avoid postoperative cancer recurrence in MDA-MB-231 breast tumors in nude mice	GdPO_4_ nanorods in the scaffold as a bioactive component enhanced stabilizing angiogenesis in calvarial defects	^112^
	Fe_3_O_4_ loaded in a polymethylmethacrylate (PMMA) bone cement scaffold	An alternating magnetic field induced magnetic nanoparticles for thermal ablation of an in situ bone tumor model	The PMMA-Fe_3_O_4_ scaffold with good mechanical support enhanced bone repair in a tibial plateau bone tumor rabbit model	^113^
Hydrogels	Hydrogenated black TiO_2_ (H-TiO_2_) coating with biomimetic hierarchical micro/nanostructures deposited on a titanium implant	Photothermal therapy induced necrosis of Sao-2 bone tumor cells in vitro and in vivo	The hierarchical micro-/nanotopography on an implant improved the adhesion, proliferation and osteogenic differentiation of BMSCs in vitro	^115^
	Polydopamine and cisplatin decorating an n-HA surface loaded in chitosan/alginate hydrogels	Photothermal therapy and chemotherapy for 4T1 breast tumor-bearing mice	The bifunctional hydrogel induced bone repair in the joint bones of rabbits	^136^
	Nanohydroxyapatite hybrid reduced graphene oxide (nHA-rGO) hydrogel	The nHA-rGO hydrogel induced photothermal therapy and killed almost all MG-63 osteosarcoma cells in vitro and in vivo	The nHA-rGO hydrogel promoted bone regeneration with the stimulation of osteoblast mineralization and collagen deposition in a rat cranial defect model	^105^
Bone-targeting nanomaterials	Gold nanorods enclosed inside mesoporous silica nanoparticles conjugated with zoledronic acid (Au@MSNs-ZOL)	Photothermal therapy enhanced by targeting to treat breast cancer bone metastasis, which was established by direct injection of MDA-MB-231 cells into the left hindlimbs of nude mice	Bone-targeting assisted inhibited the formation of osteoclast-like cells and promoted osteoblast differentiation	^167^
	Phytic acid (PA)-capped platinum (Pt) nanoparticles	Photothermal therapy of Pt nanoparticles and anticancer capabilities of PA were enhanced by tumor targeting for PC-9-Luc bone tumors in nude mice	PA/Pt nanoparticle-associated combination therapy inhibited osteolysis in the tibias of tumor-bearing nude mice	^169^

This review provides details of the recent developments in the use of bifunctional biomaterials to achieve bone tumor therapy. The new strategies in bifunctional biomaterial preparation and treatment methods are presented in the main text. Bone tumor therapy by bifunctional biomaterials is an important development direction for bone tissue engineering.^83^ Moreover, bifunctional biomaterials will play a vital role in the therapy of complex diseases, which combine tumor therapy and tissue engineering (including bone tissue engineering, skin tissue engineering, adipose tissue engineering, etc.).

## A new strategy for tumor therapy and bone regeneration

The rapid proliferation and invasion of osteosarcoma cancer cells is still the main reason why the survival rate of osteosarcoma patients has not improved in decades. Therefore, there has become an urgent need to explore new ways to treat osteosarcoma. Biomaterials for bone tumor therapy need to possess two functions: killing tumor cells and helping bone regeneration. For administration, we divided the bifunctional biomaterials into local treatment and systemic treatment options. The local bifunctional biomaterials for bone tumor therapy concentrate mainly on 3D-printed scaffolds, nano/microparticle-containing scaffolds and hydrogels. The representative systemic treatment biomaterial is bone-targeting nanoparticles for bone tumor therapy. Therefore, in this section, we present and discuss recent research on these strategies for tumor therapy and bone regeneration.

### Local treatment

#### 3D-printed scaffolds

**Fig. 2:**
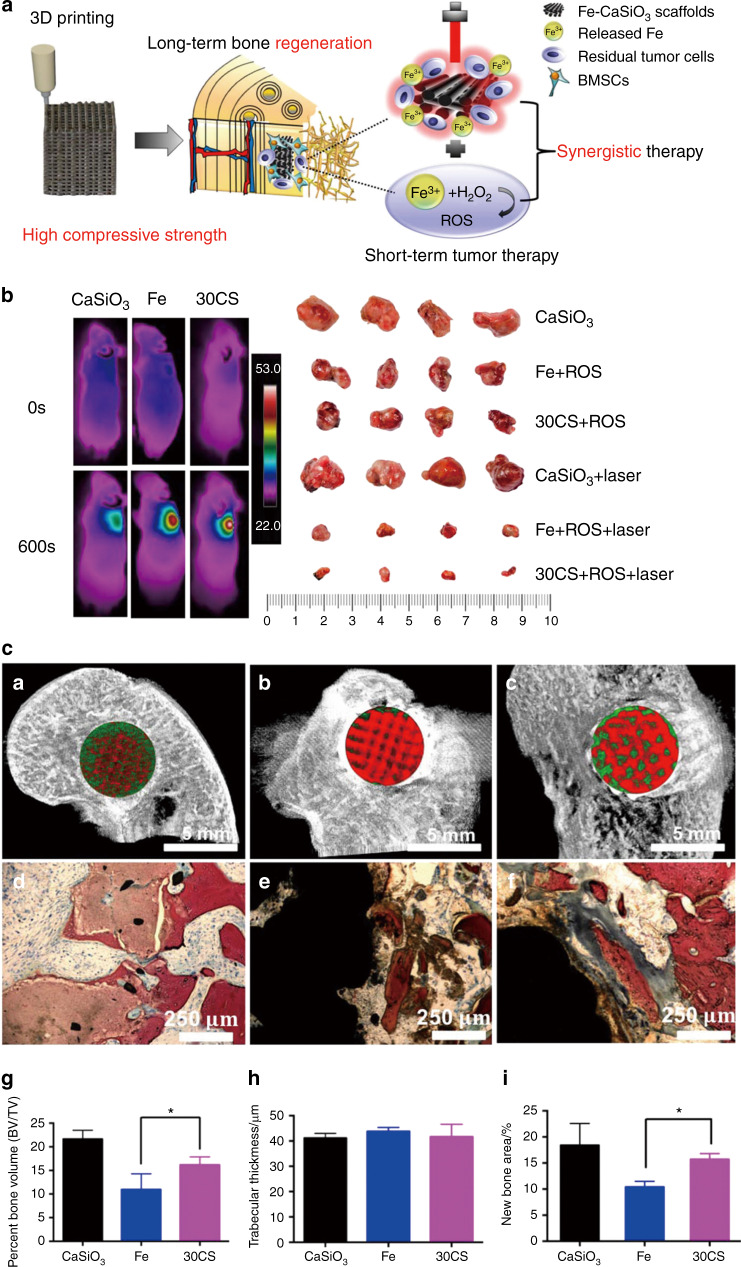
3D printing of Fe-CaSiO_3_ composite scaffolds for tumor therapy and bone regeneration. **a** The fabrication of Fe-CaSiO_3_ composite scaffolds for short-term tumor therapy and long-term bone regeneration. **b** Infrared (IR) radiation thermal images of tumor-bearing mice after irradiation with an 808 nm laser for 600 s. The photographs of the tumors from the six groups are from day 15. **c** Micro-CT images (**a**–**c**) and histological analysis (**d**–**f**) of the bone defects in the CaSiO_3_, Fe, and Fe-CaSiO_3_ (30CS) groups postsurgery in a rabbit critical-sized femoral defect model. The statistical analysis of the defects (**g**, **h**) and histomorphometric measurements of in vivo osteogenesis (**i**) in the CaSiO_3_, Fe, and 30CS groups 8 weeks post surgery. Reprinted with permission from ref. ^99^ © 2018, Nature Publishing Group

The new, innovative technology of 3D printing was first proposed by Prof. Ely Sachs.^84^ Through its rapid development, 3D printing is now widely applied in the field of tissue engineering.^85–88^ The bioactive ions in 3D-printed scaffolds, such as Ca^2+^, P^5+^, Si^4+^, Mg^2+^, Fe^3+^, and Mn^4+^, can improve osteogenic activity.^89–95^ In only a few years, a series of 3D-printed bifunctional ceramic scaffolds for tumor therapy and bone repair have been developed. Some outstanding work in this field has been done by Chengtie Wu’s group.^96,97^ For example, a 3D-printed scaffold modified with a Ca-P/polydopamine nanolayer was formulated by their group.^98^ The polydopamine nanoparticles used on the surface can cause hyperthermia to kill MDA-MB-231 tumors in nude mice. Additionally, this scaffold can release Ca and P in a sustainable manner to induce femoral defect regeneration. Moreover, a high-strength 3D bioscaffold with Fe-CaSiO_3_ was designed and prepared for tumor therapy and bone repair (Fig. 2).^99^ The 3D-printed Fe-CaSiO_3_ scaffold possessed the high compressive strength of 126 MPa, contributing to the high inherent mechanical properties of Fe. The high mechanical strength of this scaffold meets the load-bearing application requirements of human bone. Fe nanoparticles not only can provide photothermal therapy due to localized surface plasmon resonance but also can promote H_2_O_2_ decomposition to generate reactive oxygen species (ROS). Thus, synergistic photothermal and ROS therapies can enhance Saos-2 bone tumor inhibition. Furthermore, large bone defects in the legs of rabbits were repaired by an Fe-CaSiO_3_ scaffold. Recently, β-tricalcium phosphate 3D-printed scaffolds (TCP-PDLLA-LB) modified with LaB_6_ micro-nanoparticles/poly(D,L-lactide) were fabricated for tumor photothermal therapy and bone repair.^100^ Lanthanum and boron, as a “bone-seeking” element and a trace element, respectively, are bioactive, and their complex LaB_6_ possesses NIR photothermal conversion properties. Therefore, the bone tumors were significantly suppressed by photothermal therapy. Regardless of NIR laser irradiation, TCP-PDLLA-LB 3D-printed scaffolds effectively assisted in new bone formation.

In a recent example, a larnite/C 3D-printed scaffold showed an excellent photothermal effect, killing MNNG/HOS human osteosarcoma cells and inhibiting tumor growth in nude mice.^101^ Additionally, the multifunctional 3D-printed scaffold enhanced bone formation in a rat calvarial defect model. In another related study, an “all-in-one” 3D-printed biomaterial coloaded with calcium peroxide (CaO_2_) and iron oxide (Fe_3_O_4_) nanoparticles was used to solve the abovementioned dilemma in osteosarcoma therapy.^102^ The CaO_2_ produced sufficient H_2_O_2_ in the acidic tumor environment, and the Fe_3_O_4_ nanoparticles generated toxic ROS via a Fenton-like catalytic reaction. Along with magnetic hyperthermia, these two agents can produce a synergistic effect in MNNG/HOS osteosarcoma tumor-bearing BALB/c in nude mice. Importantly, the CaO_2_ nanoparticles released calcium ions to improve bone regeneration in SD rats cervical defects.

In the orthopedic field, there have been clinical trials in recent years in which 3D-printed personalized titanium plates were applied to bone defects.^103^ Inspired by their utility and encouraging clinical outcomes, Mao et al. designed and fabricated titanium plates *via* computer-aided design and computer-aided manufacturing techniques customized and fixed to the patients’ bone defects after the tumor was removed.^104^ Twelve patients with osteosarcoma had their bone tumor tissues surgically removed and were then treated with microwave-induced hyperthermia to kill the residual tumor cells. Subsequently, allograft bone and poly(methyl methacrylate) (PMMA) cement were applied to fill the bone defect. Finally, the 3D-printed personalized plate was fixed to strengthen the bone segment. Hyperthermia and 3D plate therapy improved the clinical outcomes in terms of the mean maximum flexion of the affected knees and the Musculoskeletal Tumor Society score.

#### Nano/microparticle-containing scaffolds

Nano/microparticle-containing scaffolds usually refer to inorganic-organic hybrid scaffolds. Particle-containing bifunctional hybrid scaffolds are the desired design for bone tumor therapy.^105–107^ Microspheres composed of calcium phosphate-phosphorylated adenosine were prepared with high doxorubicin (DOX) loading for bone tumor therapy.^108^ The pH-sensitive properties of microspheres presented a positive therapeutic effect on subcutaneous 143B osteosarcoma tumors in rats. Additionally, the hybrid microspheres can release active molecules to promote osteogenic differentiation in vitro. The study showed the potential application of calcium phosphate-phosphorylated adenosine microspheres for tumor inhibition and bone repair.

Additionally, a multifunctional magnetic mesoporous calcium silicate/chitosan (MCSC) porous scaffold that consisted of M-type ferrite particles (SrFe_12_O_19_), mesoporous calcium silicate (CaSiO_3_), and chitosan was prepared.^109^ The SrFe_12_O_19_ particles improved the photothermal efficacy with DOX-induced chemotherapy to reduce bone tumors. The MCSC hybrid scaffold upregulated indicators for osteogenesis. The data indicated that the MCSC hybrid scaffold promoted human bone marrow stromal cells to differentiate into osteogenic cells. In another study by the same author,^110^ fabricated SrFe_12_O_19_ nanoparticles containing bioglass/chitosan scaffolds also showed good bone repair of calvarial defects in rats.

Organic and inorganic materials are typically combined for complex disease in bone tumor treatment. Inorganic biomaterials, including nHA, TCP, bioglass, and bioceramics supply nutrients for tumor-defective bone repair. In a recent study,^111^ the surface of beta-tricalcium phosphate bioceramic (β-TCP) materials was coated with carbon aerogel, which was developed for MNNG/HOS osteosarcoma tumor therapy. The carbon aerogel coating particularly enhanced the roughness and surface area of β-TCP, resulting in good bone regeneration in a calvarial defect model.

Breast cancer-induced bone metastasis is shown to cause cancer recurrence and local bone defects. A multifunctional magnetic chitosan matrix incorporating Fe_3_O_4_ nanoparticles and GdPO_4_ nanorods was utilized for breast tumor therapy and bone defect regeneration.^112^ The Fe_3_O_4_ nanoparticles in the scaffold supplied a high temperature through photothermal effects every other day for 14 days to avoid postoperative cancer recurrence in MDA-MB-231 tumor-bearing mice. Additionally, the GdPO_4_ nanorods became orderly arranged in the scaffold and acted as a new bioactive component to induce M2 polarization of macrophages for enhanced stabilizing angiogenesis in the calvarial defect.

**Fig. 3:**
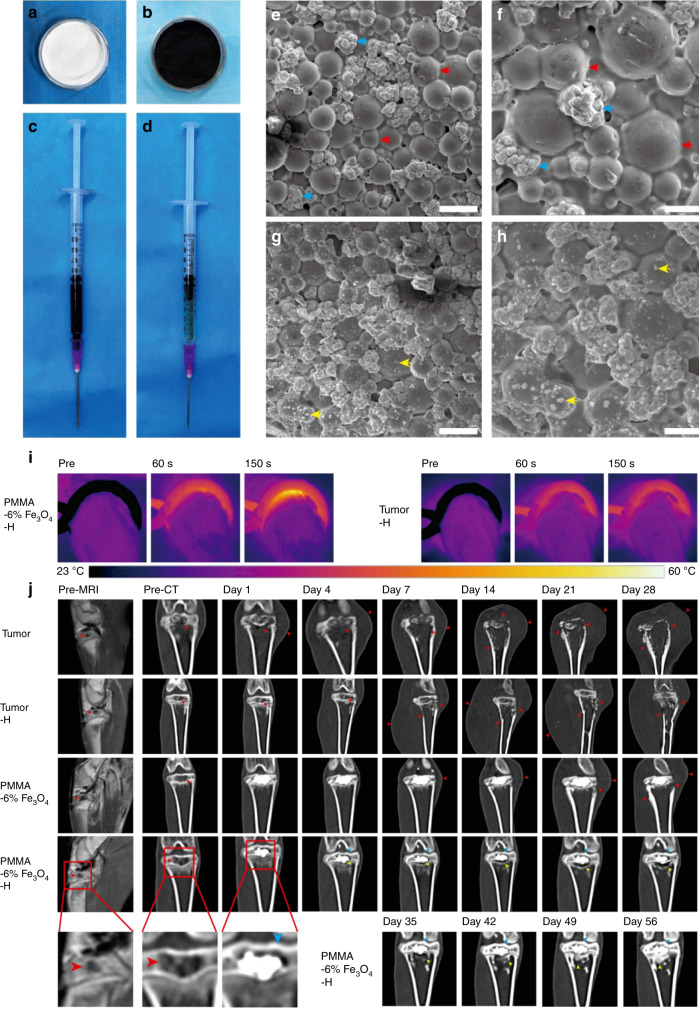
PMMA-Fe_3_O_4_ for magnetic ablation of bone tumors and bone repair. **a** PMMA powder, **b** Fe_3_O_4_ nanoparticles, **c** MMA monomer, and **d** injectable PMMA-6% Fe_3_O_4_. **e** Low-magnification SEM image of polymerized PMMA. The scale bar is 50 μm. **f** High-magnification SEM image of polymerized PMMA. The scale bar is 20 μm. **g** Low-magnification SEM image of polymerized PMMA-6% Fe_3_O_4_. The scale bar is 50 μm. **h** High-magnification SEM image of polymerized PMMA-6% Fe_2_O_3_. The scale bar is 20 μm. **i** Thermal images of rabbit legs in the PMMA-6% Fe_3_O_4_–H group and Tumor-H group. **j** Enhanced MRI images and coronal reconstructed CT images at each follow-up time point (red arrow: bone destruction and swelling of soft tissue, blue arrow: cortical bone of upper tibial plateau, yellow arrow: area of bone resorption and new bone formation). Reprinted with permission from ref. ^113^ © 2019, Ivyspring International Publisher

Another scaffold was developed from Fe_3_O_4_ magnetic nanoparticles containing PMMA bone cement with mechanical support, magnetic photothermal ablation, and bone repair features (Fig. 3).^113^ The liquid phase of these PMMA-Fe_3_O_4_ scaffolds can be accurately injected into the bone defect area. Once PMMA-Fe_3_O_4_ solidifies, an alternating magnetic field was used for the thermal ablation of the bone tumor. The fast phase transition of the PMMA-Fe_3_O_4_ scaffold prevented the leakage of Fe_3_O_4_ nanoparticles, which were nonbiodegradable during the long recovery period. Fortunately, good mechanical support is useful for physical function reconstruction. To simulate the clinical characteristics of the bone tumor, the therapeutic efficacy of the PMMA-Fe_3_O_4_ scaffold was evaluated in the tibia a tumor-bearing rabbit. The excellent heating performance provided a good VX2 tibial plateau tumor ablation outcome. The PMMA-Fe_3_O_4_ scaffold was shown to be a promising and minimally invasive agent with great clinical translation potential for the treatment of bone tumors.

Another very smart strategy is to simultaneously integrate photothermal therapy and bioactivity for bone regeneration into a single material. Bismuth (Bi)-doped bioglass provides photoinduced hyperthermia and enhanced remineralized bone tissue.^114^ The high photothermal conversion of Bi was first reported in this study. The photothermal effects were controlled by managing the radiative and nonradiative processes. Under NIR light, Bi hybrid bioglass can efficiently kill bone tumors. Moreover, Bi promotes the proliferation, differentiation, and mineralization of osteogenic cells.

Titanium is widely used in the clinical application of dental implants and is also a good choice for bone tumor therapy applications. Zheng et al.^115^ prepared a hydrogenated black TiO_2_ (abbreviated as H-TiO_2_) coating with a hierarchical porous topography on a titanium implant. The H-TiO_2_ coating surface has photothermal abilities and can induce necrosis of Saos-2 bone tumor cells. Considering that the micro/nanostructures on the implant improved the osteogenic differentiation of BMSCs, it is promising to hypothesize that BMSCs can migrate to the implant surface for bone defect regeneration. Further in vivo demonstrations of the of defect repair results are needed.

#### Hydrogels

Hydrogels are very large meshes that can contain water and have similar properties to the extracellular matrix. Hydrogels possess a highly porous structure, good biocompatibility, biodegradability, and a capability to load growth factors, leading to good bone defect repair.^116–119^ Thus, hydrogels are good candidates for bone repair. Several studies have shown potential for the use of hydrogels in bone tissue regeneration. For bone tumor therapy, the hydrogel needs to also be capable of treating tumors. It is highly advised to administer drugs or ingredients into the resected tumor area.^120,121^ Hydrogels can provide sustainable drug release for tumor illumination.^122–125^ Some hydrogels integrate interior antitumor activity with localized delivery in one system.^126^ Localized cancer treatment by hydrogels can replace the need for systemic chemotherapy administered intravenously or orally.^127–130^ With the development of multifunctional hydrogels, their applications are not limited to tissue repair but also extend to tumor cure and bone repair.

**Fig. 4:**
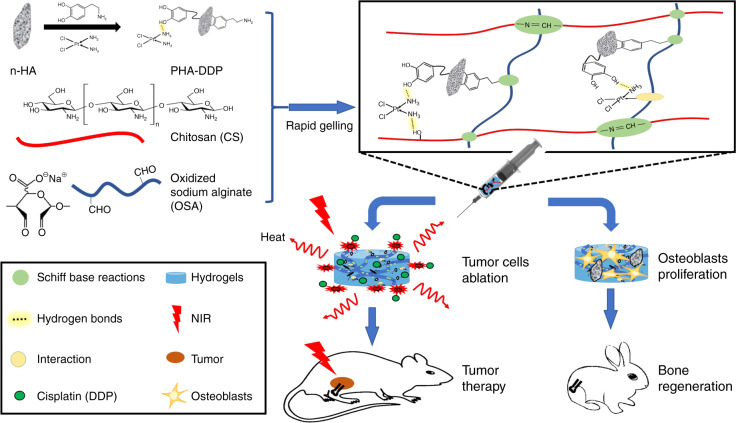
The formation of a bifunctional OSA-CS-PHA-DDP hydrogel and its bioapplication in tumor therapy and bone regeneration. Reprinted with permission from ref. ^136^ © 2019, Wiley-VCH

The ideal hydrogel system requires favorable parameters with good biocompatibility, a porous structure, adhesion to the cavity, good mechanical properties, and injectability.^131^ Among them, an injectable hydrogel can fill or match irregular defects with a mild gelation process in a minimally invasive manner.^132–135^ Recently, an injectable hydrogel was formed via a Schiff base reaction between the amino group of chitosan and the aldehyde groups of oxidized sodium alginate.^136^ As shown in Fig. 4, a hydrogel was mixed with nanohydroxyapatite (n-HA) to induce bone repair in the joint bone of a rabbit. Moreover, n-HA was decorated with polydopamine and cisplatin, which can supply photothermal therapy and chemotherapy to treat 4T1 breast tumor-bearing mice.

In another study, an in situ UV-crosslinked gelatin methacryloyl hydrogel-encapsulated liposome was formed for the local release of gemcitabine.^137^ Drug release lasts for 4 days in vitro, resulting in excellent inhibition of osteosarcoma in BALB/c mice bearing MG63 tumors. Thermosensitive hydrogels are also popular for localized drug release. For example, thermosensitive poly(L-lactide-co-glycolide)-poly(ethylene glycol)-poly(L-lactide-co-glycolide) (abbreviated as PLGA-PEG-PLGA) hydrogels were used to load DOX, methotrexate and cisplatin for localized drug delivery.^138^ Synergistic cytotoxic effects were found in the multiple drug-loaded hydrogels against osteosarcoma in vitro and in vivo. Furthermore, localized treatment caused no obvious harm to normal tissues.

A nanohydroxyapatite hybrid reduced graphene oxide (nHA-rGO) hydrogel was developed for tumor-related bone defects.^105^ The nHA-rGO hydrogel killed almost all MG-63 osteosarcoma cells via photothermal therapy. Additionally, this hydrogel promoted bone regeneration by stimulating osteoblast mineralization and collagen deposition in a rat cranial defect model.

### Systemic treatment

In recent years, there has been strong growth in nanotechnology in the fields of biology, medicine, and pharmaceuticals. Nanosized drug-based delivery platforms have been extensively studied and used for the treatment of osteosarcoma. Various nanoparticles have emerged as effective drug delivery systems in osteosarcoma treatment. Osteosarcoma tumor-invaded bone destruction contributes to an imbalance between bone reabsorption by osteoclasts and bone reconstruction by osteoblasts. Bone reabsorption promotes bone destruction and tumor metastasis processes.^139,140^ Moreover, a vicious cycle exists in osteolytic metastasis with bidirectional interactions between osteoclasts and tumor cells.^9^ Due to the low blood flow in the bone (0.05–0.20 mL·min^−1^ per gram)^141^ and blood–bone marrow barrier, targeted delivery of anticancer agents is highly recommended for bone tumor therapy. Moreover, a targeted delivery strategy shows great potential to solve systemic toxic effects and multidrug resistance, which are longstanding problems with the standard cancer chemotherapy treatment.^142,143^

Bone-modifying agents with a high affinity for bone are used for active bone targeting, including alendronate,^144–146^ zoledronic acid,^147–149^ aspartic acid,^150^ denosumab,^151^ and aptamers.^152,153^ Nanomaterials and their drug delivery systems have unique advantages for the treatment of bone tumors.^154–158^ Because bone is composed of organic matrices and inorganic minerals that are assembled at the nanoscale, and nanomaterials can assimilate into the bone microenvironment to heal diseased bone.^159,160^ Furthermore, targeted delivery systems based on nanotechnology can improve the treatment efficiency of bone tumors.^161,162^

**Fig. 5:**
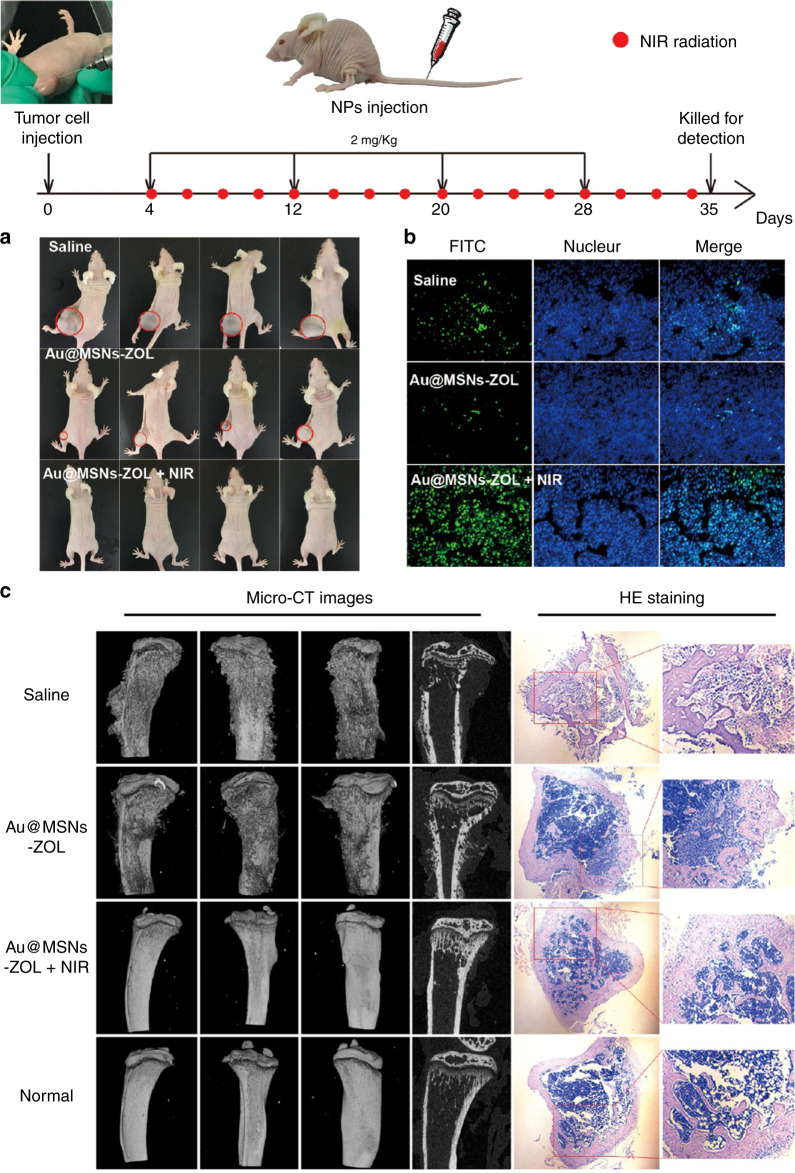
Bone-targeted nanoplatform combining zoledronate and photothermal therapy to treat breast cancer bone metastasis. Top: Timeline of the treatment schedule. A breast cancer bone metastasis mouse model was established by direct injection of MDA-MB-231 cells into the left hindlimbs of nude mice. Nanoparticles and NIR irradiation were administered as indicated. **a** Images of tumor-bearing nude mice recorded at the end of treatment (day 35). **b** TUNEL fluorescence detected in the tumor slices. **c** CT images of the tibias from different angles and micrographs of H&E-stained tibias (right). Reprinted with permission from ref. ^167^ © 2019, ACS Publications

Bisphosphonate molecules can specifically bind to the bone hydroxyapatite matrix via the chelation of calcium ions, which negatively influence osteoclast activity.^163,164^ In the 1960s, bisphosphonate was the first molecule to be identified as being able to target bone. Multifunctional melanin-like nanoparticles based on alendronate-anchored polydopamine nanoparticle hybrid Fe were reported for the bone-targeted photothermal and chemotherapy of malignant bone tumors.^165^ Alendronate possesses a high affinity for nanohydroxyapatite, resulting in targeted accumulation in the osteolytic bone site. The 7-ethyl-10-hydroxycamptothecin contained within the nanoparticles assisted with the cotherapy for efficient regression of the bone tumor. Additionally, carbon dots (CDs) synthesized from alendronate have strong binding activity for calcium-deficient hydroxyapatite.^166^ Alendronate-based CDs (Alen-CDs) showed enhanced bone targeting in the bone structures of zebrafish and rat femurs compared to nitrogen-doped CDs using ethylenediamine (Alen-EDA-CDs). These results were attributed to the bisphosphonate group on the surface of the CDs even after carbonization. Recently, gold nanorods encapsulated in mesoporous silica nanoparticles conjugated with zoledronic acid (Au@MSNs-ZOL) were prepared for bone-targeted assisted inhibition of the proliferation of osteoclast-like cells and the promotion of osteogenic differentiation (Fig. 5).^167^ The targeted photothermal therapy was enhanced to cure breast tumor bone metastasis in the hindlimbs of nude mice. This Au@MSNs-ZOL nanosystem was capable of curing the tumor, relieving pain, and inhibiting bone reabsorption for breast cancer bone metastasis treatment.

Although bisphosphonates are widely used as clinical drugs in metastatic bone tumor treatment, they may cause adverse effects such as atypical femoral fractures and esophageal cancer after long-term use.^168^ In a recent study by Cheng Yiyun’s group,^169^ phytic acid (PA)-capped platinum (Pt) nanoparticles were developed for bone-targeting therapy. PA is a natural compound that contains six phosphate groups, indicating its high bone-targeting capability. In addition, PA shows an inherent anticancer ability, which can be combined with the Pt nanoparticle photothermal therapy. An in situ bone tumor model was established by engrafting PC-9-Luc cells in the tibias of nude mice, which can be detected by imaging the luminescence of the tumor regions. After PA/Pt nanosystem treatment, PA led to an enhancement of the PA/Pt nanoparticles at the tumor site. Additionally, PA/Pt nanoparticle-associated cotherapy inhibited tumor invasion.

## Discussion and perspective

From the review on the recent development of bifunctional biomaterials in bone tumor therapy, a promising new strategy was introduced. According to the method of administration, bifunctional biomaterials can be divided into those delivered by local or systemic administration. Locally administered biomaterials mainly include 3D-printed scaffolds, nano/microparticle-containing scaffolds, and hydrogels. The representative systemically administered biomaterial is bone-targeting nanoparticles. A similarity and difference exist between these two types of biomaterials. The similarity is that a localized photothermal effect is used to kill tumor cells to prevent recurrence early on. The difference lies in the mechanism of bone repair. Locally administered scaffolds can be designed to match the bone defect area, and active molecules can be carried into the scaffold to stimulate bone regeneration. Systemically administered nanoparticles target bone tissues to inhibit bone reabsorption. The former is an example of positive regulation of bone regeneration, and the latter represents negative regulation of bone reabsorption. Although their mechanisms of bone repair are different, their outcomes in bone tumor therapy are similar.

**Fig. 6:**
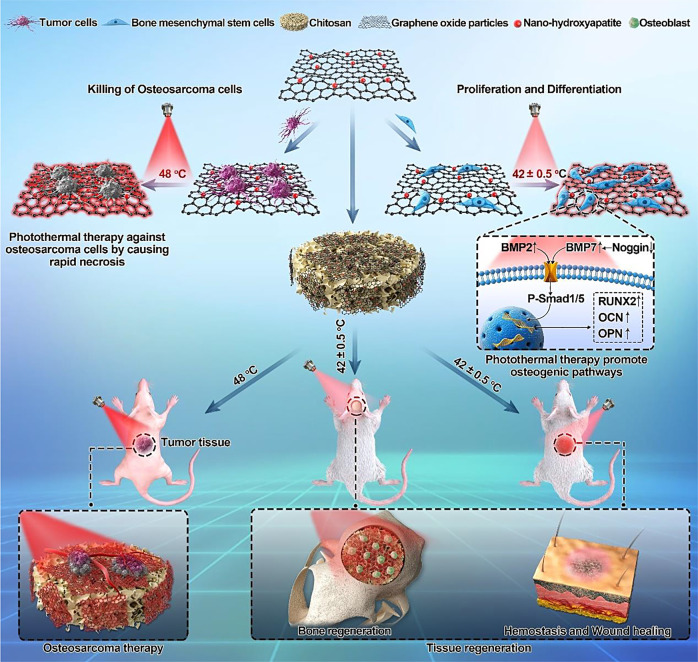
Schematic illustration of the nanohydroxyapatite/graphene oxide/chitosan (nHA/GO/CS) scaffold for osteosarcoma treatment under photothermal therapy and the promotion of tissue regeneration. Reprinted with permission from ref. ^179^ © 2020, RSC Publishing

For further development, there are three possible directions for bifunctional biomaterials for tumor therapy, and bone repair may arise. First, NIR-II window-responsive biomaterials for photothermal therapy have been developed for deep tumor treatment, as mild photothermal effects can effectively protect bone tissue. For example, a recent study reported the use of bifunctional CDs combined with WS_2_ to cure osteosarcoma under laser irradiation at 1 064 nm.^170^ Even when covered with a chicken breast with a thickness of 10 mm, the deep bone tumor was able to be killed. Moreover, mild photothermal effects (~43 °C) reported in recent studies can not only greatly enhance the proliferation of MSCs but also promote osteogenesis.^171–175^ This is good news for bone tumor therapy. The mild photothermal effect was shown to stimulate and accelerate in vitro and in vivo osteogenesis.^170,176^ Second, future treatment strategies for bone tumor therapy may not be limited to photothermal therapy and chemotherapy combined with biomaterials, and other therapeutic strategies, such as radiochemotherapy and gas therapy, may also be potential methods to treat malignant bone tumors.^171,172^ Third, scaffolds developed in the future may need to be multifunctional, considering infection along with tumor therapy and bone regeneration.^177,178^ During tumor surgery, bleeding and soft tissue defects need to be considered. As shown in Fig. 6, a nanohydroxyapatite/graphene oxide/chitosan (nHA/GO/CS) scaffold was designed to inhibit osteosarcoma growth with mild photothermal therapy and mildly high temperature (~42 °C) to promote the osteogenesis of hBMSCs.^179^ Furthermore, this scaffold showed a good hemostatic effect that can improve soft tissue regeneration.

New strategies based on bifunctional biomaterials for bone tumor therapy have the potential to extend to the treatment of other types of tumors (such as melanoma, oral tumors, and breast tumors), as well as damaged neighboring normal tissues. Melanoma treatment requires the complicated removal of tumor tissue and cutaneous defect repair.^173–175^ Oral tumors simultaneously destroy the facial bone. In clinical treatment, doctors remove oral tumor tissues along with the surrounding jawbone then collect the patient’s fibula and use it to replace the jawbone with vascular anastomosis. Breast tumor treatment involves curing the residual tumor after surgical resection and breast defect regeneration.^180^ Thus, the treatment of breast tumors relates to tumor therapy and adipose tissue engineering. In the latest study by Huang’s group, a bifunctional 3D-printed dopamine-modified alginate scaffold was used to kill breast tumors and fill the defective breast area.^23^ Researchers found a favorable photothermal effect from this scaffold to illuminate breast tumors. Additionally, the modulus of the scaffold was similar to that of normal breast tissue, and the scaffold enhanced the proliferation of breast epithelial cells. This method design can be used as a potential strategy for the prevention of breast tumor recurrence after surgery and adipose tissue repair. Future research should be focused on tumor therapy and tissue engineering for other complex diseases.

Although the use of bifunctional biomaterials for bone tumor therapy is developing rapidly, there is still a long way to go. The bifunctional biomaterial strategy further shows great potential for the treatment of complex diseases combined with tumor and tissue defects. Great confidence in researchers and clinicians will push this new strategy forward to solve clinical problems.

## Conclusion

This review highlights the recent development of bifunctional biomaterials for bone tumor therapy. A new strategy based on bifunctional biomaterials can inhibit tumor growth in the early treatment period and enhance bone repair in the late treatment period. Photothermal therapy for tumor treatment has a short duration, but bone regeneration takes a long time. With the benefits of locally administered (3D-printed scaffolds, nano/microparticle-containing scaffolds, and hydrogels) and systemically administered (bone-targeting nanoparticles) bifunctional biomaterials, the survival rate of bone tumor patients has great potential to increase. Bifunctional biomaterial treatment may provide new hope for future clinical bone tumor therapy while improving patient quality of life and decreasing mortality.

## References

[1] Siclari VA, Qin L (2010). Targeting the osteosarcoma cancer stem cell. J. Orthop. Surg. Res..

[2] Kansara M, Teng MW, Smyth MJ, Thomas DM (2014). Translational biology of osteosarcoma. Nat. Rev. Cancer.

[3] Chen D (2018). Super enhancer inbibitors suppress MYC driven transcriptional amplification and tumor progression in osteosarcoma. Bone Res..

[4] Gianferante DM, Mirabello L, Savage SA (2017). Germline and somatic genetics of osteosarcoma-connecting aetiology, biology and therapy. Nat. Rev. Endocrinol..

[5] Bosma SE, Wong KC, Paul L, Gerbers JG, Jutte PC (2018). A cadaveric comparative study on the surgical accuracy of freehand, computer navigation, and patient-specific instruments in joint-preserving bone tumor resection. Sarcoma.

[6] Friesenbichler J (2017). Clinical experience with the artificial bone graft substitute Calcibon used following curettage of benign and low-grade malignant bone tumors. Sci. Rep..

[7] Anderson ME (2016). Update on survival in osteosarcoma. Orthop. Clin. N. Am..

[8] Ballatori SE, Hinds PW (2016). Osteosarcoma: prognosis plateau warrants retinoblastma pathway targeted therapy. Signal Transduct. Target. Ther..

[9] Mundy GR (2002). Metastasis to bone: causes, consequences and therapeutic opportunities. Nat. Rev. Cancer.

[10] Weilbaecher KN, Guise TA, McCauley LK (2011). Cancer to bone: a fatal attraction. Nat. Rev. Cancer.

[11] Thibaudeau L (2014). A tissue-engineered human xenograft model of human breast cancer metastasis to bone. Dis. Models Mech..

[12] Kuchimaru T (2018). A reliable murine model for bone matastasis by injecting cancer cells through caudal arteries. Nat. Commun..

[13] Adjei IM, Sharma B, Peetla C, Labhasetwar V (2016). Inhibition of bone loss with surface-modulated, drug-loaded nanoparticles in an intraosseous model of prostate cancer. J. Control. Release.

[14] Cortini M, Baldini N, Avnet S (2019). New advances in the study of bone tumors: a lesson from the 3D environment. Front. Physiol..

[15] Ando K (2013). Current therapeutic strategies and novel approaches in osteosarcoma. Cancers.

[16] Liu Y, Yu Q, Chang J, Wu C (2019). Nanobiomaterials: from 0D to 3D for tumor therapy and tissue engineering. Nanoscale.

[17] Xue Y (2020). Engineering a biodegradable multifunctional antibacterial bioactive nanosystem for enhancing tumor photothermo-chemotherapy and bone regeneration. ACS Nano.

[18] Fan D, Tian Y, Liu Z (2019). Injectable hydrogels for localized caner therapy. Front. Chem..

[19] Zhao M (2019). Codelivery of paclitaxel and temozolomide through a photopolymerizable hydrogel prevents glioblastoma recurrence after surgical resection. J. Control. Release.

[20] Liao, J. et al. Physical-, chemical-, and biological-responsive nanomedicine for cancer therapy. *Wiley Interdiscip. Rev. Nanomed. Nanobiotechnol*. **12**, e1581 (2020). 10.1002/wnan.1581 31429208

[21] Shao J (2018). Black-phosphorus-incorporated hydrogel as a sprayable and biodegradable photothermal platform for postsurgical treatment of cancer. Adv. Sci..

[22] Du C (2017). Efficient suppression of liver metastasis cancers by paclitaxel loaded nanoparticles in PDLLA-PEG-PDLLA thermosensitive hydrogel composites. J. Biomed. Nanotechnol..

[23] luo Y (2019). 3D printing of hydrogel scaffolds for future application in photothermal therapy of breast cancer and tissue repair. Acta Biomater..

[24] Darge HF (2019). Locailized controlled release of bevacizumab and doxorubicin by thermo-sensitive hydrogel for normalization of tumor vasculature and to enhance the efficacy of chemotherapy. Int. J. Pharm..

[25] Qu Y (2015). A biodegradable thermo-responsive hybrid hydrogel: therapeutic applications in preventing the post-operative recurrence of breast cancer. NPG Asia Mater..

[26] Wang H (2019). Biocompatible iodine-starch-alginate hydrogel for tumor photothermal therapy. ACS Biomater. Sci. Eng..

[27] Chu KF, Dupuy DE (2014). Thermal ablation of tumours: biological mechanisms and advances in therapy. Nat. Rev. Cancer.

[28] Liu Y, Bhattarai P, Dai Z, Chen X (2019). Photothermal therapy and photoacoustic imaging via nanotheranostics in fighting cancer. Chem. Soc. Rev..

[29] Wang Z (2010). Construction of Bi/phthalocyanine manganese nanocomposite for trimodal imaging directed photodynamic and photothermal therapy mediated by 808 nm light. Biomaterials.

[30] Chen Q (2016). Photothermal therapy with immune-adjuvant nanoparticles together with checkpoint blockade for effective cancer immunotherapy. Nat. Commun..

[31] Zhang W (2019). A hydrogenated black TiO_2_ coating with excellent effects for photothermal of bone tumor and bone regeneration. Mater. Sci. Eng. C..

[32] Liu Y (2019). Preparation of therapeutic-laden konjac hydrogel for tumor combination therapy. Chem. Eng. J..

[33] Liao J (2017). Polymer hybrid magnetic nanocapsules encapsulating IR820 and PTX for external magnetic field-guided tumor targeting and multifunctional theranostics. Nanoscale.

[34] Zhang B, Xu C, Sun C, Yu C (2019). Polyphosphoester-based nanocarrier for combined radio-photothermal therapy of breast cancer. ACS Biomater. Sci. Eng..

[35] Hu L (2018). Oxygen-generating hybrid polymeric nanoparticles with encapsulated doxorubicin and chlorin e6 for trimodal imaging-guided combined chemo-photodynamic therapy. Theranostics.

[36] Mahmoodzadeh F, Abbasian M, Jaymand M, Salehi R, Bagherzadh-Khajehmarjan E (2018). A novel gold-based stimuli-responsive theranostic nanomedicine for chemophotothermal therapy of solid tumors. Mater. Sci. Eng. C..

[37] Liao J (2019). Magnetic/gold core-shell hybrid particles for targeting and imaging-guided photothermal cancer therapy. J. Biomed. Nanotechnol..

[38] Volsi AV (2017). Near-infrared light responsive folate targeted gold nanorods for combined photothermal-chemotherapy of osteosarcoma. ACS Appl. Mater. Interfaces.

[39] Yang S, Zhou L, Su Y, Zhang R, Dong C-M (2019). One-pot photoreduction to prepare NIR-absorbing plasmonic gold nanoparticles tethered by amphiphilic polypeptide copolymer for synergistic photothermal-chemotherapy. Chin. Chem. Lett..

[40] Liao J (2015). Combined cancer photothermal-chemotherapy based on doxorubicin/gold nanorod-loaded polymersomes. Theranostics.

[41] Yang X (2018). Gold-small interfering RNA as optically responsive nanostructures for cancer theranostics. J. Biomed. Nanotechnol..

[42] Li Q (2019). Graphene-nanoparticle-based self-healing hydrogel in preventing postoperative recurrence of breast cancer. ACS Biomater. Sci. Eng..

[43] Shi J, Kantoff PW, Wooster R, Farokhzad OC (2017). Cancer nanomedicine: progress, challenges and opportunities. Nat. Rev. Cancer.

[44] Guo X (2017). External magnetic field-enhanced chemo-photothermal combination tumor therapy via iron oxide nanoparticles. ACS Appl. Mater. Interfaces.

[45] Zhang J (2019). Multifunctional ferritin nanoparticles as theranostics for imaging-guided tumor phototherapy. J. Biomed. Nanotechnol..

[46] Farzin A, Hassan S, Emadi R, Etesami SA, Ai J (2019). Comparative evaluation of magnetic hyperthermia performance and biocompatibility of magnetite and novel Fe-doped hardystonite nanoparticles for potential bone cancer therapy. Mater. Sci. Eng. C..

[47] Du Y, Liu X, Liang Q, Liang X-J, Tian J (2019). Optimization and design of magnetic ferrite nanoparticles with uniform tumor distribution for highly sensitive MRI/MPI performance and improved magnetic hyperthermia therapy. Nano Lett..

[48] Wang K, Yang P, Guo R, Yao X, Yang W (2019). Photothermal performance of MFe_2_O_4_ nanoparticles. Chin. Chem. Lett..

[49] Liu Y (2018). The combined therapeutic effects of 131iodine-labeled multifunctional copper sulfide-loaded microspheres in treating breast cancer. Atca Pharm. Sin. B.

[50] Hu X (2017). Multifunctional CuS nanocrystals for inhibiting both osteosarcoma proliferation and bacterial infection by photothermal therapy. J. Nanopart. Res..

[51] Liu RXK (2016). An injectable self-assembling collagen-gold hybrid hydrogel for combinatorial antitumor photothermal/photodynamic therapy. Adv. Mater..

[52] Hou M (2020). Responsive agarose hydrogel incorporated with natural humic acid and MnO_2_ nanopaticles for effective relief of tumor hypoxia and enhanced photo-induced tumor therapy. Biomater. Sci..

[53] Jiang Y-W (2020). Palladium nanosheet-knotted injectable hydrogels formed via palladium-sulfur bonding for synergistic chemo-photothermal therapy. Nanoscale.

[54] Shan W (2018). Improved stable Indocyanine Green (ICG)-mediated cancer optotheranostics with naturalized hepatitis B core particles. Adv. Mater..

[55] Pan H, Zhang C, Wang T, Chen J, Sun S-K (2019). In situ fabrication of intelligent photothermal indocyanine green-alginate hydrogel for localized tumor ablation. ACS Appl. Mater. Interfaces.

[56] Cole JR, Mirin NA, Knight MW, Goodrich GP, Halas NJ (2009). Photothermal efficiencies of nanoshells and nanorods for clinical therapeutic applications. J. Phys. Chem. C..

[57] Rastinehad AR (2019). Gold nanoshell-localized photothermal ablation of prostate tumors in a clinical pilot device study. PNAS.

[58] Ni PY (2014). Injectable thermosensitive PEG-PCL-PEG hydrogel/acellular bone matrix composite for bone regeneration in cranial defects. Biomaterials.

[59] Shi R (2019). Nano twin-fiber membrane with osteogenic and antibacterial dual functions as artificial periosteum for long bone repairing. J. Biomed. Nanotechnol..

[60] Li C (2018). Glycosylated superparamagnetic nanoparticle gradients for osteochondral tissue engineering. Biomaterials.

[61] Zhang H (2017). Magnetic nanoparticle-loaded electrospun polymeric nanofibers for tissue engineering. Mater. Sci. Eng. C..

[62] Heo DN (2014). Inhibition of osteoclast differentiation by gold nanoparticles functionalized with cyclodextrin curcumin complex. ACS Nano.

[63] Chen L (2017). Drug-loaded calcium alginate hydrogel system for use in oral bone tissue repair. Int. J. Mol. Sci..

[64] Li A, Xie J, Li J (2019). Recent advances in functional nanostructured materials for bone-related diseases. J. Mater. Chem. B.

[65] Lin Y, Xiao Y, Liu C (2017). The horizon of materiobiology: a perspective on material-guided cell-behavior and tissue engineering. Chem. Rev..

[66] Yang D (2019). The immune reaction and degradation fate of scaffold in cartilage/bone tissue engineering. Mater. Sci. Eng. C..

[67] Kossover O (2020). Growth factor delivery for the repair of critical size tibia defect using an acellular, biodegradable polyethylene glycol-albumin, hydrogel implant. ACS Biomater. Sci. Eng..

[68] Ji X (2020). Mesenchymal stem cell-loaded thermosensitive hydroxypropyl chitin hydrogel combined with a three-dimensional-printed poly(Ɛ-caprolactone)/nano-hydroxyapatite scaffold to repair bone defects via osteogenesis, angiogenesis and immunomodulation. Theranostics.

[69] Lee TT (2015). Light-triggered in vivo activation of adhesive peptides regulates cell adhesion, inflammation and vascularization of biomaterials. Nat. Mater..

[70] Hou S (2019). Simultaneous nano- and microscale structural control of injectable hydrogel via assembly of nanofibrous protein microparticles for tissue regeneration. Biomaterials.

[71] Huang D (2019). Viscoelasticity in natural tissues and engineered scaffolds for tissue reconstruction. Acta Biomater..

[72] Zhang K (2018). Advanced smart biomaterials and constructs for hard tissue engineering and regeneration. Bone Res..

[73] Wu J (2019). Functionalization of silk fibroin electrospun scaffolds via BMSC affinity peptide grafting through oxidative self-polymerization of dopamine for bone regeneration. ACS Appl. Mater. Interfaces.

[74] Wang Q, Huang Y, Qian Z (2018). Nanostructured surface modification to bone implants for bone regeneration. J. Biomed. Nanotechnol..

[75] Hu Q, Liu M, Chen G, Xu Z, Lv Y (2018). Demineralized bone scaffolds with tunable matrix stiffness for efficient bone integration. ACS Appl. Mater. Interfaces.

[76] Xie J (2018). Substrate elasticity regulates adipose-derived stromal cell differentiation towards osteogenesis and adipogenesis through β-catenin transduction. Acta Biomater..

[77] Sadtler K (2016). Design, clinical translation and javascript:__doPostBack('UCCKEditor$Pane7_content$ctl18','')immunological response of biomaterials in regenerative medicine. Nat. Rev..

[78] Gu Z (2018). Double network hydrogel for tissue engineering. Wiley Interdiscip. Rev. Nanomed. Nanobiotechnol..

[79] Brokesh AM, Gaharwar AK (2020). Inorganic biomaterials for regenerative medicine. ACS Appl. Mater. Interfaces.

[80] Yao Q (2019). 3D interpenetrated graphene foam/58 S bioactive glass scaffolds for electrical-stimulation-assisted differentiation of rabbit mesenchymal stem cells to enhance bone regeneration. J. Biomed. Nanotechnol..

[81] Farokhi M (2016). Importance of dual delivery systems for bone tissue engineering. J. Control. Release.

[82] Wang W (2017). Local delivery of BMP-2 from poly(lactic-co-glycolic acid) microspheres incorporated into porous nanofibrous scaffold for bone tissue regeneration. J. Biomed. Nanotechnol..

[83] Ma H, Feng C, Chang J, Wu C (2018). 3D-printed bioceramic scaffolds: from bone tissue engineering to tumor therapy. Acta Biomater..

[84] Liu W (2013). Application and performance of 3D printing in nanobiomaterials. J. Nanomater..

[85] Matai I, Kaur G, Seyedsalehi A, McClinton A, Laurencin CT (2020). Progress in 3D bioprinting technology for tissue/organ regenerative engineering. Biomaterials.

[86] Choe G, Oh S, Seok JM, Park SA, Lee JY (2019). Graphene oxide/alginate composites as novel bioinks for three-dimensional mesenchymal stem cell printing and bone regeneration applications. Nanoscale.

[87] Yang C, Huan Z, Wang X, Wu C, Chang J (2018). 3D printed Fe scaffold with HA nanocoating for bone regeneration. ACS Biomater. Sci. Eng..

[88] Yang WF (2018). Surface-modified hydroxyapatite nanoparticle-reinforced polylactides for three-dimensional printed bone tissue engineering scaffolds. J. Biomed. Nanotechnol..

[89] Liu Y (2018). 3D-printed scaffolds with bioactive elements-induced photothermal effect for bone tumor therapy. Acta Biomater..

[90] Wang X (2017). A 3D-printed scaffold with MoS_2_ nanosheets for tumor therapy and tissue regeneration. NPG Asia Mater..

[91] Wang Y (2015). Selenite-releasing bone mineral nanoparticles retard bone tumor growth and improve healthy tissue functions in vivo. Adv. Health Mater..

[92] Pan S (2020). 2D MXene-integrated 3D-printing scaffolds for augmented osteosarcoma phototherapy and accelerated tissue reconstruction. Adv. Sci..

[93] Zheng Y, Yang Y, Deng Y (2019). Dual therapeutic cobalt-incorporated bioceramics accelerate bone tissue regeneration. Mater. Sci. Eng. C..

[94] Zhuang H (2019). Three-dimensional-printed bioceramic scaffolds with osteogenic activity for simulataneous photo/magnetothermal therapy of bone tumors. ACS Biomater. Sci. Eng..

[95] Liu G (2020). Modulating the cobalt dose range to manipulate multisystem cooperation in bone environment: a strategy to resolve the controversies about cobalt use for orthopedic application. Theranostics.

[96] Ma H (2016). A bifunctional biomaterial with photothermal effect for tumor therapy and bone regeneration. Adv. Funct. Mater..

[97] Dang W (2018). A bifunctional scaffold with CuFeSe_2_ nanocrystals for tumor therapy and bone reconstrcution. Biomaterials.

[98] Ma H (2016). 3D printing of biomaterials with mussel-inspired nanostructures for tumor therapy and tissue regeneration. Biomaterials.

[99] Ma H (2018). 3D printing of high-strength bioscaffolds for the synergistic treatment of bone cancer. NPG Asia Mater..

[100] Dang W (2019). LaB_6_ surface chemistry-reinforced scaffolds for treating bone tumors and bone defects. Appl. Mater. Today.

[101] Fu S, Hu H, Chen J, Zhu Y, Zhao S (2019). Silicone resin derived larnite/C scaffolds via 3D printing for potential tumor therapy and bone regeneration. Chem. Eng. J..

[102] Dong S, Chen Y, Yu L, Lin K, Wang X (2019). Magnetic hyperthermia-synergistic H_2_O_2_ self-sufficient catalytic suppression of osteosarcoma with enhanced bone-regeneration bioactivity by 3D-printing composite scaffolds. Adv. Funct. Mater..

[103] Xu M (2014). Custom-made locked plating for acetabular fracture: a pilot study in 24 consecutive cases. Orthopedics.

[104] Ma L (2017). 3D printed personalized titanium plates improve clinical outcome in microwave ablation of bone tumors around the knee. Sci. Rep..

[105] Li D (2018). Self-assembled hydroxyapatite-graphene scaffold for photothermal cancer therapy and bone regeneration. J. Biomed. Nanotechnol..

[106] Saber-Samandari S, Mohammadi-Aghdam M, Saber-Samandari S (2019). A novel magnetic bifunctional nanocomposite scaffold for photothermal therapy and tissue engineering. Inter. J. Bio. Macromol..

[107] Wang G (2018). A bifunctional scaffold for tissue regeneration and photothermal therapy. J. Biomed. Nanotechnol..

[108] Zhou Z-F (2017). Calcium phosphate-phosphorylated adenosine hybrid microspheres for anti-osteosarcoma drug delivery and osteogenic differentiation. Biomaterials.

[109] Yang F (2018). Magnetic mesoporous calcium sillicate/chitosan porous scafflods for enhanced bone regeneration and photothermal-chemotherapy of osteosarcoma. Sci. Rep..

[110] Lu JW, Yang F, Ke QF, Xie XT, Guo YP (2018). Magnetic nanoparticles modified-porous scaffolds for bone regeneration and photothermal therapy against tumors. Nanomedicine.

[111] Dong S (2020). A novel multifunctional carbon aerogel-coated platform for osteosarcoma therapy and enhanced bone regeneration. J. Mater. Chem. B.

[112] Zhao PP (2020). Ordered arrangement of hydrated GdPO_4_ nanorods in magnetic chitosan matrix promotes tumor photothermal therapy and bone regeneration against breast cancer bone metastases. Chem. Eng. J..

[113] Yu K (2019). PMMA-Fe_3_O_4_ for internal mechanical support and magnetic ablation of bone tumors. Theranostics.

[114] Wang L (2018). Multi-functional bismuth-doped bioglasses: combining bioactivity and photothermal response for bone tumor treatment and tissue repair. Light. Sci. Appl..

[115] Zhang W (2019). A hydrogenated black TiO_2_ coating with excellent effect for photothermal therapy of bone tumor and bone regeneration. Mater. Sci. Eng. C..

[116] Li Q (2017). The design, mechanism and biomedical application of self-healing hydrogels. Chin. Chem. Lett..

[117] Zhang K (2018). Adaptable hydrogels mediate cofactor-assisted activation of biomarker-responsive drug delivery via positive feedback for enhanced tissue regeneration. Adv. Sci..

[118] Niu Y, Yang T, Ke R, Wang C (2019). Preparation and characterization of pH-responsive sodium alginate/humic acid/konjac hydrogel for L-ascorbic acid controlled release. Mater. Express.

[119] Wang X (2018). Near-infrared light-triggered drug delivery system based on black phosphorus for in vivo bone regeneration. Biomaterials.

[120] Wu D, Xie X, Kadi AA, Zhang Y (2018). Photosensitive peptide hydrogels as smart materials for applications. Chin. Chem. Lett..

[121] Yang Z, Liu J, Lu Y (2020). Doxorubicin and CD-CUR inclusion complex co-loaded in thermosensitive hydrogel PLGA-PEG-PLGA localized administration for osteosarcoma. Int. J. Oncol..

[122] Li Q (2019). Granphene nanoparticles-based self-healing hydrogel in preventing post-operative recurrence of breast cancer. ACS Biomater. Sci. Eng..

[123] Liu Q, Wang H, Li G, Liu M, Wu H (2019). A photocleavable low molecular weight hydrogel for light-triggered drug delivery. Chin. Chem. Lett..

[124] Gumustas SA (2016). Systematic evaluation of drug-loaded hydrogels for application in osteosarcoma treatment. Curr. Pharm. Biotechnol..

[125] Hu Y, Chen X, Li Z, Zheng S, Cheng Y (2020). Thermosensitive in situ gel containing luteolin micelles is a promising efficient agent for colorectal cancer peritoneal metastasis treatment. J. Biomed. Nanotechnol..

[126] Zhao H (2020). Dual-functional guanosine-based hydrogel integrating localized delivery and anticancer activities for cancer therapy. Biomaterials.

[127] Yang Z (2018). The effect of PLGA-based hydrogel scaffold for improving the drug maximum-tolerated dose for in situ osteosarcoma treatment. Colloids Surf. B Biointerfaces.

[128] Ma H (2014). PLK1 sh RNA and doxorubicin co-loaded thermosensitive PLGA-PEG-PLGA hydrogels for osteosarcoma treatment. Biomaterials.

[129] Chen Y (2019). An injectable, near-infrared light-responsive click cross-linked azobenzene hydrogel for breast cancer chemotherapy. J. Biomed. Nanotechnol..

[130] Zheng Y (2017). Injectable hydrogel-microsphere construct with sequential degradable for locally synergistic chemotherapy. ACS Appl. Mater. Interfaces.

[131] Yu L, Ding J (2008). Injectable hydrogels as unique biomedical materials. Chem. Soc. Rev..

[132] Park MH, Joo MK, Choi GG, Jeong B (2012). Biodegradable thermogels. Acc. Chem. Res..

[133] Wasupalli GK, Verma D (2020). Injectable and thermosensitive nanofibrous hydrogel for bone tissue engineering. Mater. Sci. Eng. C..

[134] Dimatteo R, Darling NJ, Segura T (2018). In situ forming injectable hydrogels for drug delivery and wound repair. Adv. Drug Deliv. Rev..

[135] Sun Y, Nan D, Jin H, Qu X (2020). Recent advances of injectable hydrogels for drug delivery and tissue engineering applications. Polym. Test..

[136] Luo S (2019). An injectable, bifunctional hydrogel with photothermal effects for tumor therapy and bone regeneration. Macromol. Biosci..

[137] Wu W (2018). Local release of gemcitabine via in situ UV-crosslinked lipid-strengthened hydrogel for inhibiting osteosarcoma. Drug Deliv..

[138] Ma H (2015). Localized co-delivery of doxorubicin, cisplatin, and methotrexate by thermosensitive hydrogels for enhanced osteosarcoma treatment. ACS Appl. Mater. Interfaces.

[139] Roodman GD (2004). Mechanism of bone metastasis. N. Engl. J. Med..

[140] Yang Y-S (2019). Bone-targeting AAV-mediated silencing of Schnurri-3 prevents bone loss in osteoporosis. Nat. Commun..

[141] Hirabayashi H (2001). Bone-specific delivery and sustained release of diclofenac, a non-steroidal anti-inflammatory drug, via bisphosphonic prodrug based on the osteotropic drug delivery system (ODDS). J. Control. Release.

[142] Chong CR, Jänne PA (2013). The quest to overcome resistance to EGFR-targeted therapies in cancer. Nat. Med..

[143] David E (2019). 12b80-hydroxybisphosphonate linked doxorubcin: bone targeted strategy for treatment of osteosarcoma. Bioconjug. Chem..

[144] Thamake SI, Raut SL, Gryczynski Z, Ranjan AP, Vishwanatha JK (2012). Alendronte coated poly-lactic-co-glycolic acid (PLGA) nanoparticles targeting of metastatic breast cancer. Biomaterials.

[145] He Y (2017). Bisphosphonate-functionalized coordination polymer nanoparticles for the treatment of bone metastatic breast cancer. J. Control. Release.

[146] Ravanbakhsh M (2019). Mesoporous bioactive glasses for the combined application of osteosarcoma treatment and bone regeneration. Mater. Sci. Eng. C..

[147] Cole LE, Vargo-Gogola T, Roeder RK (2016). Targeted delivery to bone and mineral deposits using bisphosphonate ligands. Adv. Drug Deliv. Rev..

[148] Low SA, Kopeček J (2012). Targeting polymer therapeutics to bone. Adv. Drug Deliv. Rev..

[149] Hatami E (2019). Development of zoledronic acid-based nanoassemblies for bone-targetd anticancer therapy. ACS Biomater. Sci. Eng..

[150] Yamashita S (2018). Development of PEGylated aspartic acid-modified liposomes as a bone-targeting carrier for the delivery of paclitaxel and treatment of bone metastasis. Biomaterials.

[151] Poznak CHV (2011). American Society of Clinical Oncology executive summary of the clinical practice guideline update on the role of bone-modifying agents in metastatic breast cancer. J. Clin. Oncol..

[152] Liang C (2015). Aptamer-functionalized lipid nanoparticles targeting osteoblasts as a novel RNA interference-based bone anabolic strategy. Nat. Med..

[153] Wang Y (2017). Osteotropic peptide-mediated bone targeting for photothermal treatment of bone tumors. Biomaterials.

[154] Feng S (2019). Engineering of bone- and CD44-dual-targeting redox-sensitive liposomes for treatment of orthotopic osteosarcoma. ACS Appl. Mater. Interfaces.

[155] Yang L, Webster TJ (2009). Nanotechnology controlled drug delivery for treating bone disease. Expert Opin. Drug Deliv..

[156] Raucci MG (2019). Exfoliated black phosphorus promotes in vitro bone regeneration and suppresses osteosarcoma progression through cancer-related inflammation inhibition. ACS Appl. Mater. Interfaces.

[157] Li K (2020). Calcium-mineralized polypeptide nanoparticle for intracellular drug delivery in osteosarcoma chemotherapy. Bioact. Mater..

[158] Zhang M (2019). Fabrication of curcumin-modified TiO_2_ nanoarrays via cyclodextrin based polymer functional coatings for osteosarcoma therapy. Adv. Health Mater..

[159] Cheng H (2017). Development of nanomaterials for bone-targeted drug delivery. Drug Discov. Today.

[160] Song H (2019). Reversal of osteoporotic activity by endothelial cell-secreted bone targeting and biocompatible exosomes. Nano Lett..

[161] Rotman SG (2018). Drug delivery systems functionalized with bone mineral seeking agents for bone targeted therapeutics. J. Control. Release.

[162] Carbone EJ (2017). Osteotropic nanoscale drug delivery systems based on small molecule bone-targeting moieties. Nanomedicine.

[163] Farrell KB, Karpeisky A, Thamm DH, Zinnen S (2018). Bisphosphonate conjugation for bone specific drug targeting. Bone Rep..

[164] Chu W (2017). Calcium phosphate nanoparticles functionalized with alendronate-conjugated polyethlene glycol (PEG) for the treatment of bone metastasis. Int. J. Pharm..

[165] Wang Y (2018). Multifunctional melanin-like nanoparticles for bone-targeted chemo-photothermal therapy of malignant bone tumors and osteolysis. Biomaterials.

[166] Lee KK (2019). Bone-targeting carbon dots: effect of nitrogen-doping on binding affinity. RSC Adv..

[167] Sun W (2019). Bone-targeted nanoplatform combining zoledronate and photothermal therapy to treat breast cancer bone metastasis. ACS Nano.

[168] McClung M (2013). Bisphosphonate therapy for osteoporosis: benefits, risks, and drug holiday. Am. J. Med..

[169] Zhou Z (2019). One stone with two birds: phytic acid-capped platinum nanoparticles for targeted combination therapy of bone tumors. Biomaterials.

[170] Lu Y (2018). High-activity chitosan/nanohydroxyapatite/zoledronic acid scaffolds for simultaneous tumor inbibition, bone repair and infection eradication. Mater. Sci. Eng. C..

[171] Cojocaru FD (2019). Magnetic composite scaffolds for potential applications in radiochemotherapy of malignant bone tumors. Medicina.

[172] Yang Q (2020). Engineering 2D mesoporous silica@MXene-integrated 3D-printing scaffolds for combinatory osteosarcoma therapy and NO-augmented bone regeneration. Small.

[173] Yu Q (2018). Copper silicate hollow microspheres incorporated scaffolds for chemo-photothermal therapy of melanoma and tissue engineering. ACS Nano.

[174] Zhou L (2019). Injectable self-healing antibacterial bioactive polypeptide-based hybrid nanosystems for efficiently treating multidrug resistant infection, skin-tumor therapy, and enhancing wound healing. Adv. Funct. Mater..

[175] Li J (2019). Lysozyme-assisted photothermal eradiction of methicillin-resistant Staphylococcus aureus infection and acceletated tissue repair with natural melanosome nanostructures. ACS Nano.

[176] Yanagi T, Kajiya H, Kawaguchi M, Kido H, Fukushima T (2015). Photothermal stress triggered by near infrared-irradiated carbon nanotubes promotes bone deposition in rat calvarial defects. J. Biomater. Appl..

[177] Lu Y (2018). High-activity chitosan/nanohydroxyapatite/zoledronic acid scaffolds for simultaneous tumor inhibition, bone repair and infection eradication. Mater. Sci. Eng. C..

[178] Lu Y (2018). Zero-dimensional carbon dots enhance bone regeneration, osterosarcoma ablation, and clinical bacterial eradication. Bioconjug. Chem..

[179] Ma L (2020). A novel photothermally controlled multifunctional scaffold for clinical treatment of osteosarcoma and tissue regeneration. Mater. Today.

[180] Wang X (2018). Bifunctional scaffolds for the photothermal therapy of breast tumor cells and adipose tissue regeneration. J. Mater. Chem. B.

